# *Caragana korshinskii* Kom. plantation reduced soil aggregate stability and aggregate-associated organic carbon on desert steppe

**DOI:** 10.7717/peerj.12507

**Published:** 2022-02-16

**Authors:** Qi Lu, Hongbin Ma, Yao Zhou, Roberto Calvelo-Pereira, Yan Shen

**Affiliations:** 1 Ningxia University, School of Agricultural, Yinchuan, Ningxia, China; 2 Ningxia University, Breeding Base for State Key Laboratory of Land Degradation and Ecological Restoration of Northwest China, Yinchuan, Ningxia, China; 3 Massey University, School of Agriculture and Environment, Palmerston North, New Zealand

**Keywords:** Caragana shrubs, Desert steppe grassland, Soil aggregate stability, Aggregate-associated organic carbon

## Abstract

**Background:**

After implementing of the “Grain-for-Green” project, *Caragana korshinskii* Kom. has been widely planted in China’s arid regions. Although natural restoration grassland and artificial *Caragana* plantations measures have long been focuses in carbon research, the combined influence of natural restoration grassland and artificial *Caragana* plantation measures on aggregate stability and the aggregate-associated organic carbon (OC) remains unclear.

**Method:**

We selected natural grassland (NG) and three different densities of *Caragana* plantations (high planting density, HG; middle planting density, MD; low planting density, LD) on desert steppe. The soil aggregate distribution and stability index such as fractal dimension (D), mean weight diameter (MWD), geometric mean diameter (GMD), percentage of aggregation destruction (PAD), as well as aggregate-associated OC concentration and stock were measured.

**Results:**

Results shows that the soil aggregates were primarily macroaggregates (>2 mm) and mesoaggregates (0.25–2 mm) under dry sieving while microaggregates (<0.25 mm) were preponderant under wet sieving (more than 57%). Overall, compared with *Caragana* plantations, the MWD (4.43 and 4.51 mm) and GMD (1.72 and 1.83 mm) were both highest in two soil layers under the NG and the D (2.77 and 2.71) was lowest. Compared with the NG, the aggregate-associated OC stocks in the 0–40 cm depths in the LD, MD, and HD decreased by 41.54%, 46.93%, and 42.03%, respectively. SOC stock was mainly concentrated in the soil aggregate with sizes of >2 mm and <0.25 mm. These results suggested that natural grassland restoration measures could improve the soil aggregate stability and aggregate-associated OC concentration better than *Caragana* plantation restoration measures, which NG may be optimal for increasing carbon sequestration and stabilizing soil aggregates on desert steppe.

## Introduction

The desert steppe of China’s arid regions in China is part of the world that suffers from severe soil erosion (Fu et al., 2011) and is a very fragile ecological environment (Chen et al., 2007; Wei et al., 2010) where soil erosion control has become very relevant. Due to excessive human interference with the natural ecosystem, the ecosystem is becoming increasingly fragile, leading to desertification of grasslands and severe soil erosion. As an essential part of the terrestrial ecosystem, vegetation is the center of material circulation and energy flow in the ecosystem and plays a vital role in soil and water conservation and carbon sequestration. The growth of vegetation can effectively improve soil structure, input more organic matter into the soil system, and improve soil quality (Dou et al., 2020). It has been reported that vegetation restoration can control soil erosion and improve ecological environmental conditions (Xu et al., 2014). Reforestation has become a strategic decision to address the environment (Fu et al., 2011). Since 1999, China has implemented the “Grain for Green Program” (GGP), which is one of the most comprehensive ecological reconstruction programs (State Forestry Administration, 1999–2011), also known as the Returning Farmland to Forests or Grassland Project (Deng, Liu & Shangguan, 2014). Although the arid region’ desert steppe has been improved in terms of soil and water conservation (Fu et al., 2011), including wind erosion reduction, improved sand fixation (Wang, Shao & Shao, 2010), and increasing the carbon storage after large-scale plantations (Jia et al., 2017), the suitability of various vegetation restoration methods remains controversial (Jiao et al., 2012). This is due to differences in climate, soil and vegetation, causing significant variability in China’s arid and semiarid regions (Gao et al., 2014). However, despite the number of studies focusing on ecosystem restoration is increasing, how soil stability is modified by vegetation restoration remains a poorly understood process.

Over the last two decades, several researchers have examined the responses of soil aggregate size distribution and stability to management measures, and reported the role of aggregates in soil organic carbon accumulation. Zhu, Shangguan & Deng (2017) indicated that natural restoration grasslands had better soil organic carbon and aggregate stability than plant forests on the Loess Plateau, China. Cheng et al. (2015) also revealed that after vegetation restoration, macroaggregates’ content increased significantly, enhancing the uniformity of the soil aggregate size distribution and inducing greater soil organic carbon sequestration. However, these studies did not integrate soil physical and chemical property indicators to comprehensively evaluate aggregates’ stability and explore the relationship between soil aggregate stability parameters and the organic carbon of each size class of the aggregates. Soil aggregates are soil structural units with a diameter of <10 mm formed by the rearrangement, flocculation and cementation of soil particles (Bronick & Lal, 2005). Aggregates are usually grouped by size: macroaggregates (>2 mm; Qiu et al., 2015; Wei et al., 2013), mesoaggregates (0.25–2 mm; Li et al., 2007), and microaggregates (<0.25 mm; Tisdall, 1996; Shrestha et al., 2004). New vegetation establishment accelerates the cementation of soil particles and redistributes aggregates of different size, ultimately determining the magnitude and direction of soil C accumulation (Qiu et al., 2015). A number of researchers have demonstrated that the macroaggregates had larger SOC concentration accumulation and higher soil aggregates stability. Similarly, it has been reported that macroaggregates and mesoaggregates were a source of organic carbon enrichment (Puget P.Angers D.A.Chenu, 1998; Puget P.Chenu C.Balesdent, 2000). However, some researchers had the distinctive standpoints, for instance, Christensen (1986) research demonstrated that microaggregates have also been proven to be the primary contributor to soil carbon sequestration. Therefore, it is essential to clarify the concentration of different aggregate size fractions in driving changes of SOC concentration. The hierarchical theory of aggregation proposed that microaggregates form mesoaggregates and macroaggregates (Edwards & Bremner, 1967), with organic matter contributions as a binding agent (Haynes & Swift, 1990). Research has suggested that, in certain soils, increases in aggregate stability are associated with the storage of more soil organic carbon (Haynes & Beare, 1997). Moreover, the permanence of carbon inside microaggregates impacted long-term soil carbon accumulation (Six & Paustian, 2014). In arid and semiarid environments, both wind and water erosion significantly impacted the soils (Okolo et al., 2020). The stability of dry stable aggregates (DSA) can be used to evaluate the ability of soil to resist wind erosion effects, while the stability of wet stable aggregates (WSA) is more suitable for predicting the ability of soil to resist rainfall erosion (Okolo et al., 2020). Parameters commonly used to study structure and aggregate stability in soils include soil mean weight diameter (MWD), geometric mean diameter (GMD), percentage of aggregate destruction (PAD), and fractal dimension (D) (Zhou et al., 2020). Large values of MWD and GMD indicate higher average particle size class of soil aggregates and better soil structure stability (Zhu, Shangguan & Deng, 2017). The larger the fractal dimension (D) value of soil aggregates, the higher the possibility of aggregate breakage and the gradual increase in the number of microaggregates in the soil (Castrignano & Stelluti, 1999).

*Caragana korshinskii* is a legume shrub widely planted in the arid desert steppe areas of China. *Caragana* is a pioneer plant with rapid growth and high resistance to drought, cold, and barrenness, thereby used to control grassland soil erosion and avoid desertification (Ma et al., 2008; Fang et al., 2008). In recent years, ecological construction projects, including *Caragana* shrubs’ planting, have been under development on desert grasslands, such as those in Ningxia, Inner Mongolia, and Gansu in the eastern area of the Loess Plateau of China (Gao et al., 2014). Recent research had mainly focused on the effects of vegetation rehabilitation on the distribution of aggregates and aggregate-associated OC (Fu et al., 2009; Kirkels, Cammeraat & Kuhn, 2014; Zhou et al., 2007) and the dynamics of soil carbon sequestration (Zhu, Shangguan & Deng, 2017). In addition, the effect of planting *Caragana* shrubs on parameters such as soil nutrients and stoichiometries in this region have been reported (Yang & Liu, 2019). However, few studies have investigated the different impacts on the two land uses (natural restoration grassland and man-made *Caragana* shrubs plantations) on soil aggregate stability and aggregate-associated OC in the desert steppe of the arid region of China. Therefore, an improved understanding of soil aggregate stability and aggregate-associated OC in *Caragana* plantations in the arid desert steppe is necessary. To propose a theoretically based rational design for the restoration method of desert steppe, we assumed that (1) aggregate stability and aggregate-associated OC would be more favorable in the soil under natural grassland than in *Caragana* shrub-land and (2) SOC in macroaggregates and mesoaggregates would be positively associated with aggregate stability. In this study, we investigated the effects of *Caragana* shrubs established at three planting densities (HD, high planting density; MD, middle planting density; LD, low planting density) on the soil aggregate stability and soil aggregate stability parameters on the soil aggregate organic carbon of different soil aggregate size classes. Therefore, the objectives of this study are (1) to analyze the soil aggregate fraction distribution and soil aggregate stability in natural grasslands and at different *Caragana* planting densities; (2) to determine the distribution of SOC associated with the size fractions of aggregate classes; and (3) to investigate the relationship between soil aggregate stability parameters and the aggregate organic carbon of different soil aggregate size classes. Finally, these results can provide a basis of further assessing of *Crargana* shrub planting measures in the arid desert steppe of China or other similar regions.

## Materials and Methods

### Experimental site

**Figure 1:**
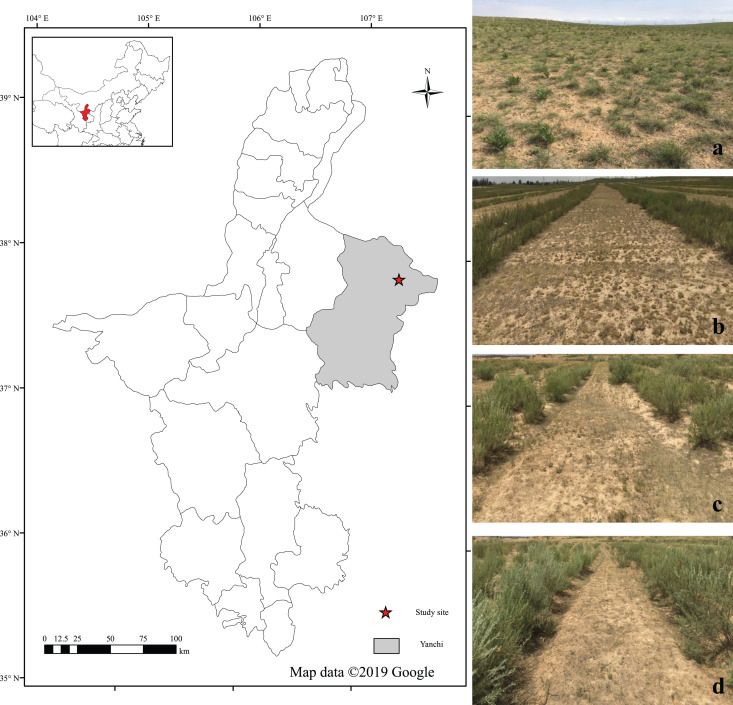
Location of the study area in the Yanchi County, Ningxia, China. The photos of the grasslands were (A) natural grassland, (B) low *Caragana* planting density grassland, (C) middle *Caragana* planting density grassland and (D) high *Caragana* planting density grassland. (Map created by ArcGIS Desktop 10.5, Esri, Redlands, CA, US. https://desktop.arcgis.com; Photo credit: Qi Lu).

The field experimental site is located on the southern edge of the Mu Us Desert, in the arid desert steppe of Yanchi County (107°19′E, 37°88′N), located in Northwest China (Fig. 1). The area is characterized by a typical temperate continental arid climate, with an annual average temperature of 7.6 °C, annual accumulated temperature ≥ 0 °C of 3,430 °C, mean annual precipitation of 290 mm, and average annual evaporation of 2,132 mm. The soil type is dominantly desertification sierozem, based on Chinese Soil Taxonomy (Soil Survey Staff, 2010). Throughout the year, alternating strong northwesterly winter and spring winds and heavy summer rainfall, leading to the region suffer severe wind and water erosion.

At the background of “Grain for Green Program” implementation, many *Caragana* shrub and natural restoration grasslands were distributed in the study area, and the *Caragana* population recruitment was generally realized by reproduction from seed. At the study site, large numbers of *Caragana* shrubs have been planted and fenced since 2003. *Caragana* shrubs are distributed on the desert steppe in strips with different densities. Through 17 years vegetation restoration, the main dominant species are *Lespedeza davurica*, *Leymus secalinus*, *Artemisia scoparia*, *Oxytropis psamocharis*, *Euphorbia esula*, and *Corispermum mongolicum*.

### Experimental design

Experimental site zonal vegetation belongs to desert steppe. *Caragana* shrubs are distributed on the desert steppe in strips with different densities, including 4,690 bundles/hm^2^ (HD, high planting density), 3,573 bundles/hm^2^ (MD, middle planting density), and 2,012 bundles/hm^2^ (LD, low planting density). *Caragana* inter-shrub grasslands with consistent topography, soil, vegetation, and growth conditions were selected as the study plots in the experimental area. There is a large area where *Caragana* shrubs are not planted which is considered NG (natural grassland). The slope, aspect, elevation and other natural factors were carefully considered when plots were selected to ensure that their topography features were roughly consistent. The main soil physical and chemical properties of the study sites are shown in Table 1. *Caragana* shrubland was paired with adjacent natural restoration grassland to ensure the two restoration types had similar land use history. Then, soil aggregate stability and organic carbon distribution were studied among three *Caragana* shrub (HD, MD, and LD) planting densities and natural grassland (NG). Finally, the characteristics of *Caragana* and the ground grassland vegetation between *Caragana* shrub belts at each site are listed in Table 2. The schematic diagram of natural grassland and different densities of *Caragana* planting is shown in Fig. 1.

**Table 1:** Basic soil characteristics of *Caragana korshinskii* plantations (0–40 cm).

Site	Bulk density (g/cm3)	Soil organic carbon (g/kg)	Total soil nitrogen (g/kg)	Alkali-hydrolyzale nitrogen (mg/kg)	Total soil phosphorus (g/kg)	Available phosphorus (mg/kg)	Soil water content (%)
NG	1.49 ± 0.01ab	4.49 ± 0.46a	0.08 ± 0.01ab	15.94 ± 1.68ab	0.31 ± 0.01a	2.52 ± 0.15b	6.82 ± 0.43a
LD	1.50 ± 0.01ab	4.81 ± 0.40a	0.07 ± 0.01b	18.89 ± 1.43a	0.27 ± 0.01b	2.96 ± 0.12ab	2.70 ± 0.30b
MD	1.45 ± 0.02b	5.62 ± 0.50a	0.10 ± 0.01a	17.00 ± 1.14ab	0.27 ± 0.01b	3.37 ± 0.20a	3.05 ± 0.30b
HD	1.50 ± 0.02a	6.46 ± 1.66a	0.07 ± 0.01ab	14.18 ± 0.99b	0.25 ± 0.01b	3.25 ± 0.22a	2.91 ± 0.25b

**Note:**

Different letters in the same column indicate significant differences at the 0.05 level. NG, natural grassland; LD, low planting density; MD, middle planting density; HD, high planting density.

**Table 2:** The ground grassland vegetation characteristics between *Caragana* shrubs belts.

Site	*Caragana* shrubs			Ground grassland vegetation			
	Planting density (Cluster/hm^2^)	Height (cm)	Shrub biomass (kg/ha)	Density (Plants/m2)	Height (cm)	Coverage (%)	Herbaceous biomass (g/m^2^)
NG	–	–	–	130.00 ± 5.86bc	10.51 ± 0.72a	51.67 ± 2.19a	72.58 ± 6.41a
LD	2,012	150.43 ± 4.81a	2644.93 ± 4.61b	210.33 ± 1.52a	7.24 ± 0.36b	37.00 ± 3.51b	37.62 ± 2.70bc
MD	3,573	121.53 ± 0.64b	3378.60 ± 6.39a	165.00 ± 1.26b	6.82 ± 0.21b	42.67 ± 3.71ab	44.96 ± 4.43b
HD	4,690	110.30 ± 3.10b	1590.49 ± 9.12c	106.67 ± 3.76c	5.94 ± 0.49b	24.33 ± 2.73c	29.04 ± 2.22c

**Note:**

Different letters in the same column indicate significant differences at the 0.05 level. NG, natural grassland; LD, low planting density; MD, middle planting density; HD, high planting density.

### Experimental sampling

The soil sampling was conducted in early August 2019. Three sampling plots (50 m × 50 m) were chosen at random on the desert grasslands between each *Caragana* shrub density (*i.e*., HD, MD, LD) and natural grassland (NG). At the center and four corners of each plot, five 1 m × 1 m quadrats were chosen to obtain soil bulk density and undisturbed soil samples at depths of 0–20 cm and 20–40 cm, respectively, and then sealed in a plastic box to avoid being crushed and impacted during transportation back to the laboratory. A soil drilling sampler was used to sample the 0–20 cm and 20–40 cm soil layers of each plot. Five soil samples were taken from the center and four corners of each plot, and then the five auger samples were pooled to make a composite sample at each depth for the measurement of soil physical and chemical properties. In the laboratory, the samples were air-dried at room temperature and stored for further analyses.

### Analyses of soil physical and chemical properties

#### Separation of soil aggregates

The stability of soil aggregates was determined using conventional dry and wet sieving methods (ISSAS, 1978). A 0.5 kg air-dried soil samples were passed through a nest of flat sieves 5, 2, and 0.25 mm in sequence using a dry-sieving method, and the soil aggregates in each sieve was weighed to determine the ratio of different aggregate components to the total soil mass. The total weight of the soil aggregates was determined by adding the weights of the soil aggregates in the four size sections (>5, 2–5, 0.25–2, and <0.25 mm).

The aggregates at all levels determined by the dry sieve were mixed into 50 g air-dried soil samples according to the ratio. After pouring the prepared soil sample on the sieve group (2, 0.5, and 0.25 mm), the sieve group was immersed in water for 10 min (Kemper, Rosenau & Nelson, 1985). Then, the screen was shaken up and down slowly 30 times and removed. The soil samples on the sieves of all levels were washed into a beaker with water and then oven-dried at 40 °C for 48 h to constant weight. The soil bulk density (BD) was measured using the ring knife method (Hossain, Chen & Zhang, 2015).

#### Chemical characterization of soil samples

The Kjeldahl method (Bremner, 1960) was utilized to analyze total soil nitrogen (TN). Soil total phosphorus (TP) was established using the molybdophosphate method after wet digestion with H_2_SO_4_ (Parkinson & Allen, 1975). Available nitrogen (AN) was determined using a microdiffusion technique after the samples were subjected to alkaline hydrolysis (Wang, Liu & Xue, 2012). The soil extract available phosphorus (AP) was determined by sodium bicarbonate extraction (Olsen et al., 1954). Soil organic carbon (SOC) was determined using dichromate oxidation (Walkley & Black, 1934).

#### Study of fractal dimension

The fractal dimension (D) of the soil aggregates was studied following the equation:

(1) $$(3 - D)\lg (di/d\max ) = \lg (W_{(\delta \lt di)}/W_0)$$

where *W*_*(δ< di)*_ is the cumulative mass of soil particles smaller than d_*i*_ and W_0_ is the sum of the masses of all the grain size particles. Using this model, lg(*d*_*i*_*/d*_*max*_) and lg(*W*_*(δ< di)*_ */W*_*0*_) were used as the horizontal and vertical coordinates, and the fractal dimension was calculated by the regression method (Turcotte, 1986; Chakrabortia et al., 2003).

#### Assessment of soil aggregate stability

To assess the impact of different treatments (NG, HD, MD and LD) on soil structure, we calculated two indexes related to soil aggregate stability: mean weight diameter (MWD) and geometric mean diameter (GMD) (Klute, Kemper & Rosenau, 1986). The MWD and GMD were calculated using the following equations (Chaplot & Cooper, 2015; Obalum & Obi, 2014):

(2) $$MWD = \sum\limits_{i = 1}^n {X_iW_i}$$

(3) $$GMD = \exp (\displaystyle{{\sum\limits_{i = 1}^n {(\ln X_i)W_i} } \over {\sum\limits_{i = 1}^n {W_i} }})$$

where *n* is the number of fractions (>5, 3–5, 2–3, 1–2, 0.5–1, 0.25–0.5, <0.25 mm), *X*_*i*_ is the mean diameter (mm) of the sieve size class (5, 3, 2, 1, 0.5, and 0.25 mm), and *W*_*i*_ is the proportion of the soil retained on the sieve.

Additionally, the percentage of aggregate destruction (PAD, %) was calculated as:

(4) $$PAD = \displaystyle{{W{\rm - }W^\prime} \over W} \times 100$$

where *W* is the mass fraction of aggregates >0.25 mm after dry sieving; and *W*′ is the mass fraction of aggregates >0.25 mm after wet sieving.

#### The stock of OC in bulk soil and aggregate

The stock of OC (g m^−2^) in bulk soil calculated using the following equation:

(5) $$SOC_{BS} = \displaystyle{{H \times BD \times OC} \over {100}}$$

where *BD* is the soil bulk density (g cm^−3^), *H* is the thickness (cm) of the soil layer, and *OC* is the OC content (g kg^−1^) in different soil layers.

The stocks of OC (g m^−2^) associated with each size fraction were calculated as follows:

(6) $$SOC_{A{\rm i}} = \displaystyle{{H \times BD \times OC_i \times W_i} \over {10}}$$

where *OC*_*i*_ is the OC content (g kg^−1^) associated with each aggregate size fraction.

### Statistical Analyses

**Figure 2:**
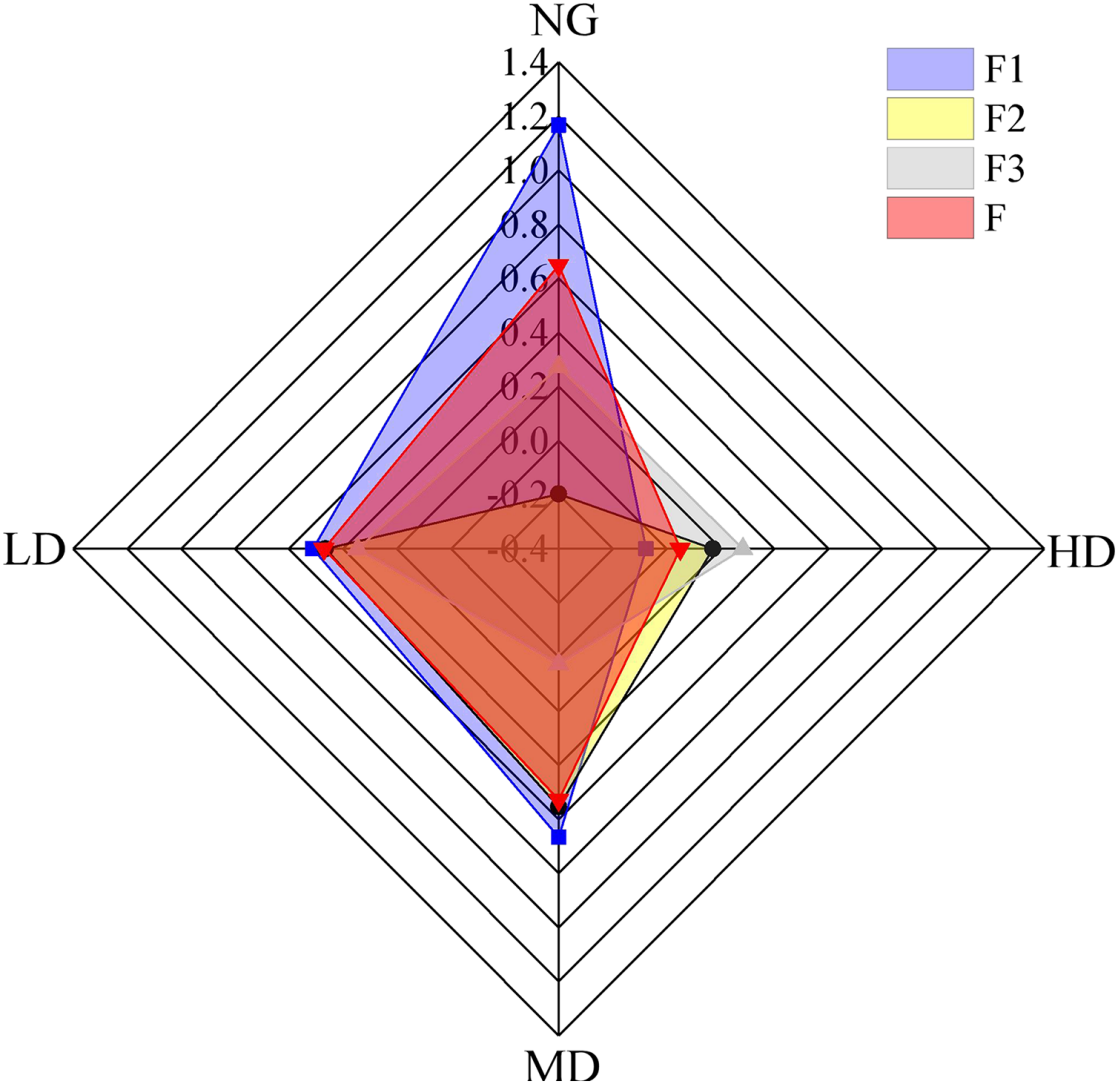
Comprehensive scores of different *Caragana* planting densities. NG, natural grassland; LD, low planting density; MD, middle planting density; HD, high planting density.

One-way analysis of variance (ANOVA) was conducted using SPSS software (Version 19.0) to compare aggregate size distribution, percentage of aggregate destruction (PAD), mean weight diameter (MWD), geometric mean diameter (GMD), and fractal dimension (D). Multiple comparisons of means for each variable were performed using a least significant difference (LSD) at a significance level (α) = 0.05. We chose the related indicators, including soil properties (BD, SOC, TN, TP, AN, and AP), D, PAD, MWD, and GMD, as the initial variables to perform principal component analysis. Then, we selected the common factors, F1, F2, and F3 by the factors analysis method in SPSS based on these related indicators. Finally, using this method, we calculated the total score (F value) of soil aggregate stability. According to these scores, we drew the Fig. 2 in our manuscript. General linear regression models (GLRMs) were used to evaluate the influence of soil aggregate stability on the soil organic carbon of different soil aggregate size classes. The graphs of the proportion of different aggregate fractions, the MWD, GMD, and D of soil aggregate and PAD value under different vegetation restoration measures were drawn by Microsoft Excel 2010. The radar charts were created with Origin 9.0 (OriginLab Corporation, Northampton, MA, USA).

## Results

### Aggregate size distribution

**Figure 3:**
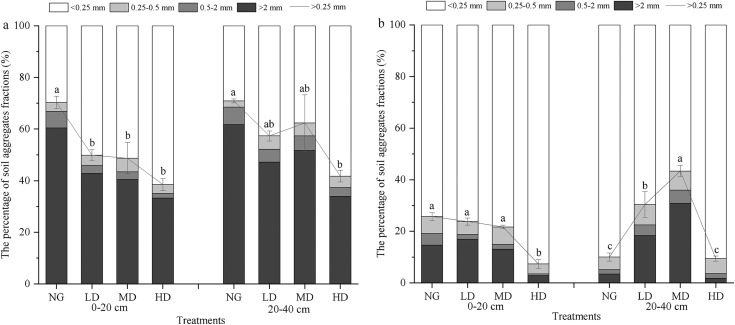
Dry sieving (A) and wet sieving (B) aggregate contents of different fractions at 0–40 cm depth of soil in different *Caragana* planting densities. NG, natural grassland; HD, high planting density; MD, middle planting density; LD, low planting density. The values for the >0.25 mm aggregate in different land use types followed by the different lowercase letters indicate significant difference (*P* < 0.05).

The soil aggregate contents were varied across *Canagana* planting densities (Fig. 3). At a depth of 0–20 cm, NG = 60%, HD = 33%, MD = 40%, and LD = 43% of the >2 mm dry sieving aggregate were observed (Fig. 3A). Samples collected from 20–40 cm depth, the percentage of >2 mm aggregates for the four treatments increased in the following order: NG > MD > LD > HD (Fig. 3A). In detail, at soil depths of 0–20 cm, the content of >0.25 mm aggregates in the NG, MD, and LD treatments was significantly higher than that in HD (*P* < 0.05). Moreover, under the NG and MD treatments, the content of >0.25 mm aggregates in the 20–40 cm soil was significantly higher than that of HD and LD (*P* < 0.05). The soil of mid-planting density had significantly more material in the >0.25 mm size class relative to the other *Caragana* treatments, although natural grassland soil had more macroaggregates (*P* < 0.05) (Fig. 3A).

Under each treatment 0–40 cm soil layer, the wet sieving aggregates are dominated by a particle size of <0.25 mm, with content of 57–93%, followed by >2 mm particle size, and the content of 0.5–2 mm water-stable aggregates is the lowest of 1–5% (Fig. 3B). At the 0–20 cm soil depth, there was no significant difference in the content of aggregates >0.25 mm between treatments (*P* > 0.05). However, at a soil depth of 20–40 cm, the content of >0.25 mm aggregates under the MD treatment was significantly higher than that of the other treatments (*P* < 0.05).

### Stability parameters (D, MWD, GMD, and PAD) of soil aggregates planted with different Caragana planting densities

**Figure 4:**
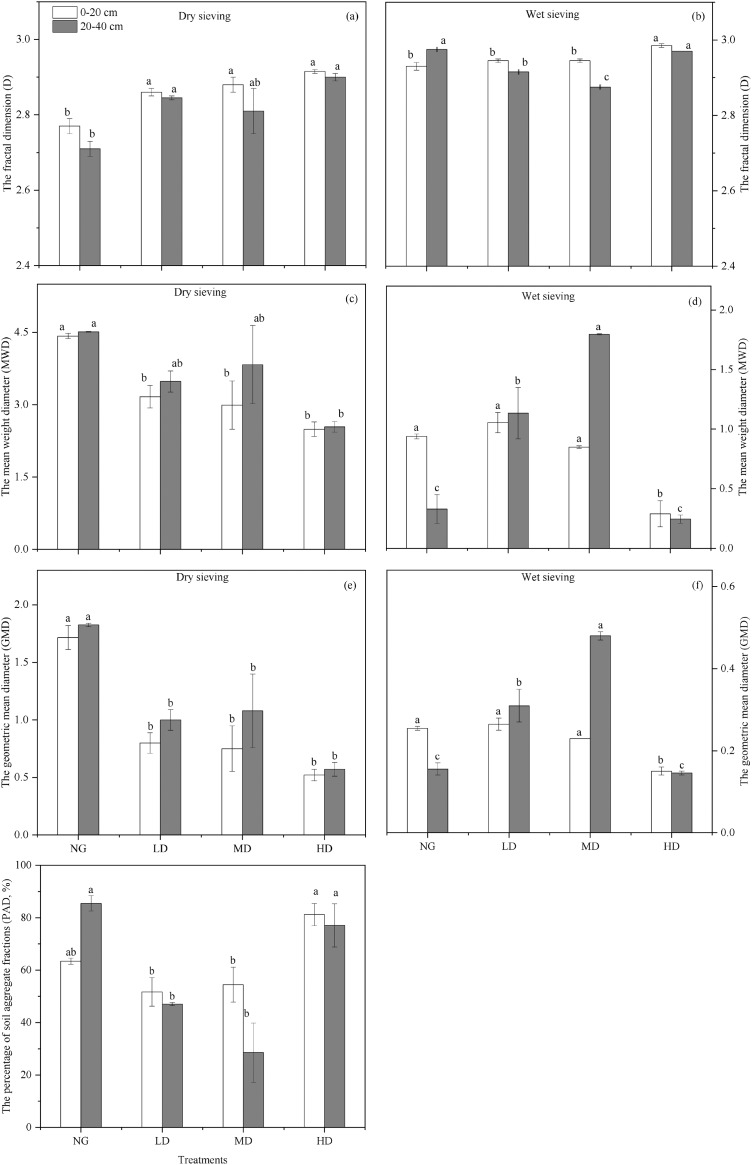
The D (A), MWD (mm) (B), GMD (mm) (C), and PAD (%) (D) values from 0–40 cm of soil aggregate in different *Caragana* planting densities in sample sites on the Loess in desert steppe. Different lowercase letters correspond to significant difference at *P* < 0.05. The bars represent standard errors. NG, natural grassland; HD, high planting density; MD, middle planting density; LD, low planting density. D, the fractal dimension; MWD, the mean weight diameter; GMD, the geometric mean diameter; PAD, the percentage of aggregate destruction.

Regression analysis was used to calculate the fractal dimension D value of the soil aggregate particle size at different *Caragana* planting densities (Fig. 4A). The D value of each treatment ranged from 2.71 to 2.99 in the 0–20 cm soil layer. For dry-sieving aggregate, the D value in the NG treatment was significantly smaller (*P* < 0.05) than that in the other treatment. For wet-sieving, treatment HD was significantly larger (*P* < 0.05) than the other treatments (Fig. 4A). The same variation trend was observed for the aggregates at the 20–40 cm soil depth.

The results show that the value of MWD and GMD of the dry stable aggregates obtained by the dry-sieving method are higher than those of the wet-sieving method (Fig. 4). The mean weight diameter of dry-sieving aggregates (D-MWD) and the geometric mean diameter of dry-sieving aggregates (D-GMD) of the NG treatment were significantly larger than those of the *Caragana* plantation. In terms of the three types of *Caragana* plantation sites, the D-GMD and W-GMD values of the LD treatment at soil depths of 0–20 cm and 20–40 cm were significantly larger than those of the other treatments (*P* < 0.05). The LSD test showed that at a soil depth of 20–40 cm, the W-MWD and W-GMD of the MD treatment were significantly larger than those of the NG and several *Caragana* plantation treatments (*P* < 0.05). Additionally, the W-GMD of wet-sieving aggregates and its change rule of W-MWD are consistent.

Our results showed that the percentage of aggregate destruction (PAD) varied on average from 29–85% (Fig. 4). The PAD of HD >0.25 mm aggregates in the 0–20 cm soil depth was significantly higher than that in the other treatments (81%) (*P* < 0.05). At 20–40 cm soil depths, MD was significantly lower than that of the NG and HD treatments (*P* < 0.05), and the PAD of the >0.25 mm aggregate in the MD reached a minimum (29%). For all of the sites, PADs of different plantation distances demonstrated the following order: HD (79%) > NG (74%) > LD (42%) > MD (49%).

### The comprehensive score of soil aggregate stability

The soil dry aggregate size distribution is mainly affected by wind erosion, so statistical software was used to perform principal component analysis on 11 soil texture and dry aggregate index indicators and extract four principal components (PCs) (Table 3). The principal component eigenvalues were 4.56, 2.17, and 1.38 (both > 1) and explained 74% of the data variability. Therefore, the first three principal components are extracted. According to the results of the principal component analysis, the initial factor coefficient loading matrix can be obtained, and combined with the variables of the standardization processes, the expressions of the principal components are obtained as follows:

**Table 3:** Contribution of factorial loads and eigenvalues of the analyzed variables of soil texture and dry aggregate indexes indicators.

Indexes	Factors			Coefficient matrix		
	1	2	3	1	2	3
*X1*	0.87	−0.35	0.03	0.41	−0.24	0.02
*X2*	0.90	−0.31	0.07	0.42	−0.21	0.06
*X3*	0.85	−0.43	0.05	0.40	−0.29	0.04
*X4*	0.22	0.75	−0.29	0.10	0.51	−0.24
*X5*	−0.33	−0.03	−0.78	−0.15	−0.02	−0.67
*X6*	0.73	0.44	−0.07	0.34	0.30	−0.06
*X7*	−0.57	−0.45	0.54	−0.27	−0.30	0.46
*X8*	−0.36	0.40	0.33	−0.17	0.27	0.28
*X 9*	0.92	0.00	0.02	0.43	0.00	0.02
*X10*	0.52	0.63	0.24	0.24	0.43	0.20
*X11*	0.03	0.49	0.47	0.01	0.33	0.40
Eigenvalue	4.56	2.17	1.38			
Variance (%)	41.41	19.69	12.56			
Cumulative variance (%)	41.41	61.09	73.66			

**Note:**

PC, Principal component; *X*1, D-D, fractal dimension by dry-sieving; *X*2, D-MWD, mean weight diameter by dry-sieving; *X*3, D-GMD, geometric mean diameter by dry-sieving; *X*4, PAD, percentage of aggregate destruction; *X*5, SOC, soil organic carbon; *X*6, TN, total nitrogen; *X*7, BD, soil bulk density; *X*8, AP, available Phosphorus; *X*9, TP, total phosphorus; *X*10, AN, available nitrogen; *X*11, D-WR_0.25_ = >0.25 mm dry-sieving aggregate specific gravity.

$\eqalign{
  & {\rm{F1  = 0}}{\rm{.41X1 + 0}}{\rm{.42X2 + 0}}{\rm{.40X3 + 0}}{\rm{.10X4 - 0}}{\rm{.15X5 + 0}}{\rm{.34X6 - 0}}{\rm{.27X7 - 0}}{\rm{.17X8 }}  \cr 
  & \,\,\,\,\,\,\,\,{\rm{ + 0}}{\rm{.43X9 + 0}}{\rm{.24X10 + 0}}{\rm{.01X11;}}  \cr 
  & {\rm{F2 =  - 0}}{\rm{.24X1 - 0}}{\rm{.21X2 - 0}}{\rm{.29X3 + 0}}{\rm{.51X4 - 0}}{\rm{.02X5 + 0}}{\rm{.30X6 - 0}}{\rm{.30X7  }}  \cr 
  & {\rm{ }}\,\,\,\,\,{\rm{ +  0}}{\rm{.27X8 + 0}}{\rm{.00X9 + 0}}{\rm{.43X10 + 0}}{\rm{.33X11;}}  \cr 
  & {\rm{F3 = 0}}{\rm{.02X1 + 0}}{\rm{.06X2 + 0}}{\rm{.04X3 - 0}}{\rm{.24X4 - 0}}{\rm{.67X5 - 0}}{\rm{.06X6 + 0}}{\rm{.46X7  }}  \cr 
  & \,\,\,\,\,{\rm{  +  0}}{\rm{.28X8 + 0}}{\rm{.02X9 + 0}}{\rm{.20X10 + 0}}{\rm{.40X11}}{\rm{.}} \cr} $

The first, second, and third principal components explain 41.41%, 19.69%, and 19.69% of the variability, respectively. Therefore, the weights of the first, second, and third principal components in the sum of the three principal components are 56.22%, 26.73%, and 17.05%, respectively. The formula for comprehensive evaluation can be obtained: F = 0.562F1 + 0.267F2 + 0.171F3. The comprehensive evaluation index is obtained by linear weighted summation to analyze the stability of soil aggregates. The larger the value, the more stable the soil aggregates. Figure 2 comprehensively evaluates the stability of soil aggregates at different *Caragana* planting densities. The results showed that at a soil depth of 0–20 cm, the desert grassland scored the highest, while at a soil depth of 20–40 cm, the grassland with middle *Caragana* planting density had the highest comprehensive score. Overall, the comprehensive score of the entire 0–40 cm soil layer was: NG > MD > LD > HD.

### Aggregate-associated OC concentration and stock

**Figure 5:**
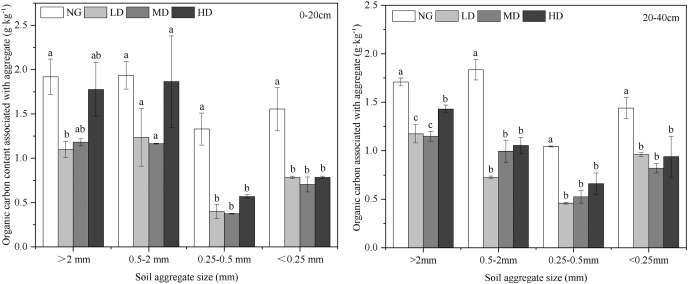
Distribution of wet stable aggregate-associated OC concentration at 0–40 cm depth of different *Caragana* planting densities (g kg^−1^). Different lowercase letters correspond to significant difference at *P* < 0.05. The bars represent standard errors. NG, natural grassland; HD, high planting density; MD, middle planting density; LD, low planting density.

Figure 5 shows that the average organic carbon concentration in aggregates of different sizes ranged between 0.38 and 1.94 g C/kg soils. The SOC concentration of each aggregate fraction has the highest carbon content in >2 mm aggregates and the lowest organic carbon in the 0.25–0.5 mm aggregates. The soil aggregate-associated OC concentration in the NG was significantly higher than that of any treatment (*P* < 0.05). In the 0–20 cm and 20–40 cm soil layers, for the treatments of *Canagana*, the aggregate-associated OC concentrations were the highest in the HD treatment, ranging from 0.57 to 1.87 g/kg. In the same soil layer for the same treatment, the aggregate-associated OC concentrations with different fractions varied slightly, and the mesoaggregates (0.25–2 mm) had high OC concentrations.

**Figure 6:**
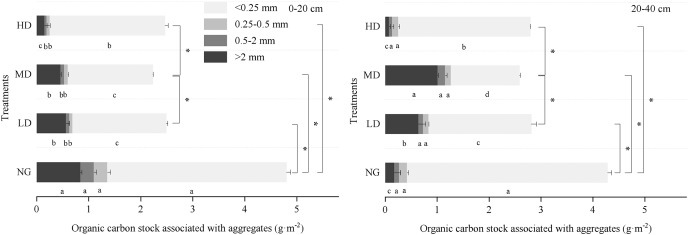
Aggregate-associated OC stock in different *Caragana* planting densities. Lowercase letters indicate the differences in SOC in the aggregates with different sizes in the same treatments (*P* < 0.05). *Significant difference (at the 0.05 level) in soil aggregate-associated OC stock in different Caragana planting densities in the 0–20 and 20–40 cm soil layers.

The highest OC stock associated with macroaggregate, mesoaggregate, and microaggregate was found in NG, both at 0–20 and 20–40 cm depth (Fig. 6). The aggregate-associated OC stock was mainly concentrated in >2 and <0.25 mm aggregates. From the perspective of soil depth, the aggregate-associated OC stock of the shrubs with different planting densities increased in deeper soil layers, while the aggregate-associated OC stock in the natural grassland decreased with the depth of the soil layer.

### Relationship between aggregate stability and the SOC of different aggregate fractions

**Figure 7:**
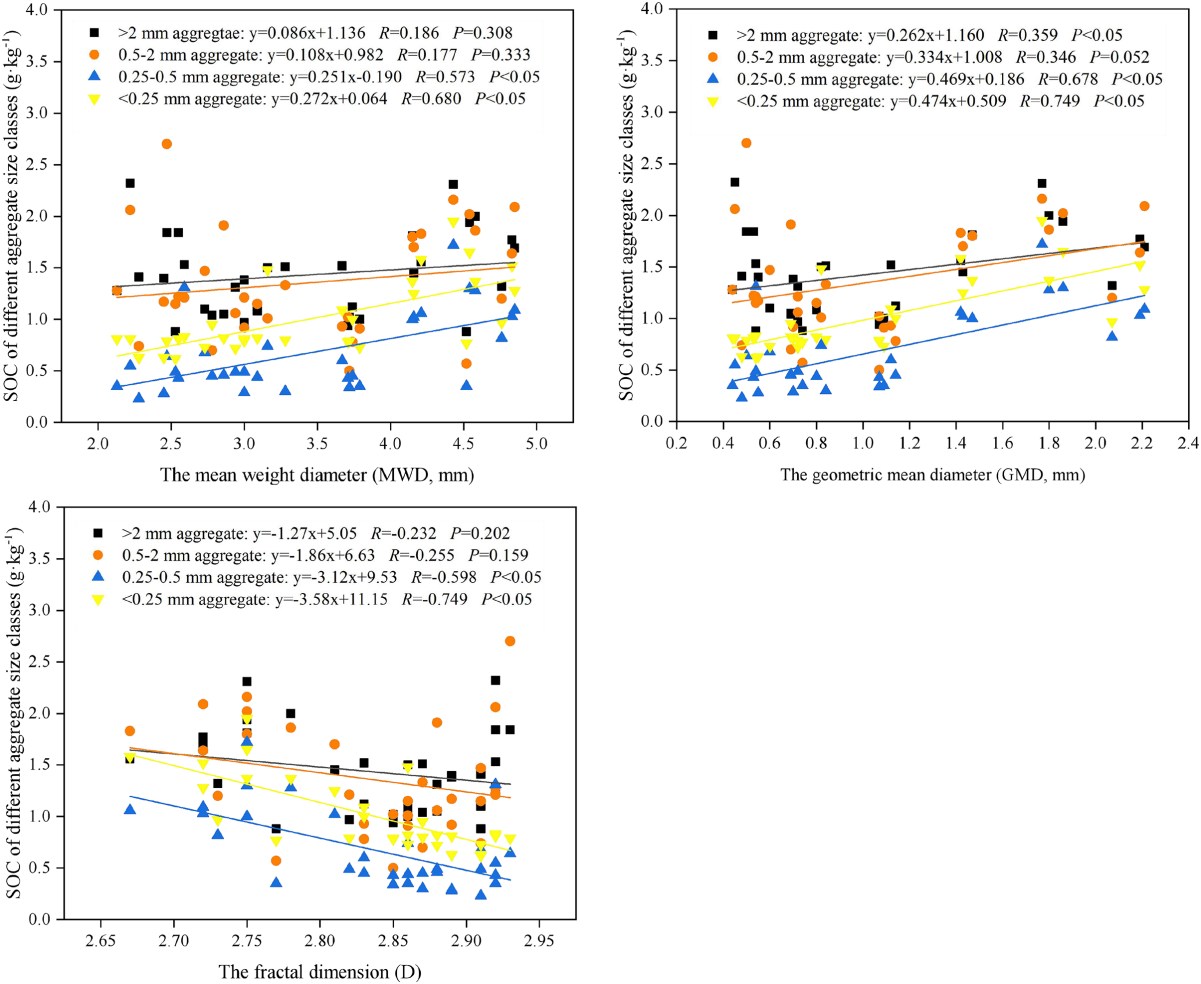
Relationships between aggregate-associated carbon (g kg^−1^) and soil aggregate stability (MWD-w, Mean weight diameter of dry sieving aggregates, mm; GMD-w, Mean weight diameter of wet sieving aggregates, mm; and D-w, Fractal dimension of wet siev). Statistical models are general linear regression model with SOC of different aggregate size classes as an independent variable and soil aggregate stability parameters (MWD, GMW, and D) as fixed factor.

The SOC concentration in the 0.25–0.5 mm and microaggregates (<0.25 mm) were significantly positively correlated with MWD and GMW, and significantly negatively correlated with D (*P* < 0.05) (Fig. 7). The SOC concentration in the large macroaggregates (>2 mm) was significantly and positively correlated with GMW (*P* < 0.05).

## Discussion

In this study, the organic carbon content in aggregates and its relationship with the aggregates’ stability was analyzed. Our results suggested that whether *Caragana* plantations can improve soil aggregates’ stability and the accumulation of SOC depends on the *Caragana* shrub planting density. Compare to natural grassland and *Caragana* shrub plantation, the natural restoration of grasslands can be beneficial to promote the formation of soil aggregates and aggregate-associated OC.

### Distribution of the soil aggregate stability index between the dry and wet sieve methods in the two land-use types

Generally, land use and soil management affect soil aggregate size distribution and stability (Hu et al., 2015). Soil aggregate stability can be used as one indicator of soil quality evaluation (Arshad & Cohen, 1992). The stability of soil aggregates affects soil characteristics, including soil porosity, compactness, aeration, and erosion resistance (Six, Elliott & Paustian, 2000). We found that the aggregate stability of the surface soil in the natural desert steppe plot was better than that of the *Caragana korshinskii* plantation plots (Fig. 4). Table 2 showed that the height, coverage and grassland aboveground herbaceous biomass on the natural desert steppe are significantly higher than those on the grassland where *Caragana* is planted. Therefore, the impact of litter accumulation in the natural desert steppe is large, which is conducive to the accumulation of soil nutrients (Pérès et al., 2013). Furthermore, the lignin and cellulose from plant litter bring more binding agents, such as polysaccharides and fungi, which increase soil aggregates’ stability (Zeng et al., 2020). In contrast, in the subsurface soil layer, aggregates’ stability in the *Caragana* low planting density plot was better than that of the natural desert steppe plot (Fig. 4). It is well known that established reforestation can have a profound impact on soil. For example, the introduction and growth of exotic shrub species may change the composition of the original local vegetation community (Zhang et al., 2020), thereby increasing the potential for carbon sequestration and affecting soil aggregates’ stability (Cavagnaro, Cunningham & Fitzpatrick, 2016). Soil water content is an essential limiting factor in arid desert grassland ecosystems. The amount of water input to the desert steppe is very small and largely unpredictable. Therefore, the fiercest competition among vegetation communities in arid regions is the competition for water (Noy-Meir, 1973). Similarly, in the present study, the soil water content of the shrub-grown desert steppe was significantly lower than that of the natural desert steppe (Table 1). The stability of deep grassland soil structure after shrub planting in arid areas may be closely related to the planting density of shrubs. High-density planting of *Caragana* may not be suitable for ecological restoration in arid areas because it would cause severe water shortages in deep soils (Zhang et al., 2020).

The root system is another important factor affecting the formation and stability of soil aggregates. A previous study reported that the roots of herbaceous plants are mainly fine roots, while *Caragana* belongs to shrubs, which have thicker roots. Gyssels et al. (2005) reported that fine roots can increase the direct contact area between roots and soil, which is more conducive to enhancing the soil aggregates’ stability. Compared with areas where *Caragana* is planted, bare grassland soil is not tilled, and herbaceous vegetation contributes to the aggregation of fine soil particles by root exudates and biomass and by adding organic material into the soil (Qiu et al., 2015). In natural grasslands where *Caragana* is not planted, the root system is mostly an herbaceous root system, which is shallower in the soil. Undisturbed soil may promote fungal growth and the proliferation of fungal hyphae that contribute to macroaggregate formation (Beare et al., 1993). The cementation of polysaccharides and humus in soil organic matter on soil particles can improve soil stability (Bai & Zhou, 2020). For the desert steppe that grows *Caragana*, many root systems that penetrate into the soil can mechanically destroy existing aggregates (Hu et al., 2019).

The fractal dimension (D) is not only one of the parameters reflecting soil stability but also an alternative indicator to describe the desertification process (Gao et al., 2014). Perfect & Kay (1991) characterized the soil aggregate size distribution of different cropping treatments by fractal theory. The average mass diameter (MWD) and average geometric diameter (GMD) of soil aggregates are commonly used indicators to reflect soil aggregates’ size distribution. The larger the MWD and GMD values are, the higher the average particle size of the aggregate and the higher the stability (Celik, 2005). The results indicated that after *Caragana* shrub belts were planted in the desert steppe, soil macroaggregates were more easily disrupted, and aggregates’ stability declined. In addition, it can be seen from Fig. 2 that the effects of the middle planting density *Caragana* land on the stability of the soil aggregate were the greatest, the low planting density *Caragana* land was second, and the high planting density *Caragana* land was the worst compared to the natural desert steppe. In addition, in the middle-density *Caragana* planting area, the biomass of *Caragana* shrubs was significantly greater than that of shrubs with low and high planting densities (Table 2). This means that there were large amounts of litter and organic matter in the soil of the middle-density *Caragana* planting area; thus, the concentration of soil nutrients was higher (Zhang et al., 2018). Furthermore, the MWD and GMD values of wet-sieving aggregates were smaller than those of dry-sieving aggregates. The reason is that many non-water-stable aggregates are decomposed, indicating that there were many dried soil aggregates in this area (Zhou et al., 2020). Therefore, in terms of this result of soil aggregate stability, we can conclude that undisturbed grassland can improve the stability parameters of aggregates more effectively than afforestation. Compared with the restoration of natural desert grasslands, this study emphasizes the positive impact of middle-density *Caragana* planting on soil aggregates’ stability.

### The influence of natural grassland and Caragana planting density on soil aggregate-associated carbon

We can comprehensively and objectively understand the changes in the soil organic carbon pool (Bai, Zhou & He, 2020). Planting woody species in arid areas may promote soil carbon accumulation in the soil (Zhou, Boutton & Wu, 2017). The effects of planting shrubs on soil carbon concentrations in arid and semi-arid areas have been studied in the past, but the results have been mixed. Some studies found a significant increase in soil OC concentration following the revegetation of desert steppe (Su et al., 2010; Wang et al., 2019), whereas Cunningham et al. (2012) found that three decades of afforestation did not lead to substantial changes in the carbon concentration of the soil. It is speculated that on a longer time scale, shrub plantings are likely to have larger impacts on the amount and forms of soil carbon (Wang et al., 2019). Our results showed that shrubland exhibited a higher soil organic carbon content than natural grassland, but the aggregate-associated OC concentration was lower than that of natural grassland (Table 1) (Fig. 5). This may be related to the short cultivation period of *Caragana*, which did not have a significant impact on the soil aggregates. The high concentration of aggregates in natural desert steppe areas could explain the relatively high aggregate stability observed in the soils (Fig. 4). This could be due to the absence of tillage of the natural desert steppe. This can also be attributed to the different carbon sequestration potentials of grass and shrubs (Guo, Wang & Gifford, 2007). Lignified litter enters the soil and turns into organic matter at a slower rate than herb litter (Paul et al., 2017). In addition, the establishment and development of *Caragana* shrubs may disturb and accelerate the decomposition of litter (Yang, Liu & An, 2018). The sum of aggregate-associated OC concentrations in NG was 2.8 and 2.4 g/kg^−1^ higher than those in the *Caragana* shrub plantation area at depths of 0–20 cm and 20–40 cm, respectively (Fig. 5), indicating that natural grasslands have greater carbon sequestration potential than artificial *Caragana* shrubs (Zhong et al., 2021).

The dynamic change in SOC not only depended on the input of organic matter but was also closely related to the structure of the soil aggregates (Zhang et al., 2016). Moreover, based on four dominant land-use types on the Loess Plateau, Zhong et al. (2019) revealed that the physical and chemical protection of organic carbon in aggregates is one of the main mechanisms of carbon sequestration in soil. In addition, our study showed that the increase in SOC concentration overtime was more dependent on macroaggregates and mesoaggregates than on microaggregates. Here, we have confirmed that mesoaggregate fractions have the highest organic carbon concentration (Fig. 5). Fresh residues first enter the soil and form microaggregates, which are then encrusted with intra-aggregate particulate organic matter and microbial products to form macroaggregates (Six, Elliott & Paustian, 2000). Fungi dominated the macroaggregates and mesoaggregates to a greater extent than the other fractions. Bacteria enrichment is often reported for microaggregate fractions (Smith et al., 2014). Bacterial cell walls are more susceptible to decomposition than fungal cell walls. The decomposition rate of fungal secretions is slow, and the mean residence time in the soil is long (Guggenberger et al., 1999; Six et al., 2006). Therefore, the mesoaggregates and macroaggregates provided better physicochemical protection to the organic carbon associated with these fractions.

## Conclusions

We evaluated the effects of different *Caragana* shrub planting densities and natural restoration grasslands on soil aggregate stability and aggregate-associated carbon in the desert steppe of an arid region of China. *Caragana* plantations destroyed the macroaggregate and mesoaggregate fraction structure of desert steppe soil with a concomitant reduction in soil aggregate-associated OC, whereas natural grassland favored soil aggregate-associated OC accumulation. The comprehensive soil aggregate stability scores are ordered as follows: NG > MD > LD > HD. However, due to the high content of microaggregates, the retention of SOC during *Canagana* plantation and natural restoration can be attributed to the accumulation of OC in microaggregates. Overall, natural restoration grassland had a better effect than planting *Caragana* shrubs in terms of improving the soil structure and increasing the soil aggregate-associated OC concentration.

## Supplemental Information

10.7717/peerj.12507/supp-1 Supplemental Information 1 Raw data.

## Additional Information and Declarations

### Data Availability

The following information was supplied regarding data availability: The raw measurements are available in the Supplemental Files.
